# Differentiation of benign and malignant breast lesions by ultrasound localization microscopy

**DOI:** 10.1186/s13244-025-02013-6

**Published:** 2025-06-18

**Authors:** Jia Li, Cong Wei, Tao Ying, Yan Liu, Ronghui Wang, Maoyao Li, Chao Feng, Di Sun, Yuanyi Zheng

**Affiliations:** 1 Department of Ultrasound in Medicine, Shanghai Sixth People’s Hospital Affiliated to Shanghai Jiao Tong University School of Medicine, Shanghai, China; 2 Department of Ultrasound, Shanghai Ninth People’s Hospital, Shanghai Jiao Tong University School of Medicine, Shanghai, China; 3 Department of Ultrasound in Medicine, Dali Bai Autonomous Prefecture People’s Hospital, Yunnan, China; 4 Department of Ultrasound, Ruijin Hospital, Shanghai Jiao Tong University School of Medicine, Shanghai, China; 5 Division of andrology, Department of reproductive medicine, The International Peace Maternity and Child Health Hospital, School of Medicine, Shanghai Jiao Tong University, Shanghai, China; 6 Division of andrology, Department of reproductive medicine, Shanghai Sixth People’s Hospital Affiliated to Shanghai Jiao Tong University School of Medicine, Shanghai, China

**Keywords:** Ultrasound localization microscopy, Breast lesions, Microvasculature, Contrast-enhanced ultrasound

## Abstract

**Objective:**

We investigated the role of ultrasound localization microscopy (ULM) qualitative and quantitative parameters in distinguishing benign from malignant breast lesions.

**Methods:**

The ULM qualitative and quantitative parameters of breast lesions were recorded. A receiver operating characteristic (ROC) curve was applied to assess the diagnostic performance of ULM. Intra- and inter-operator reliabilities of quantitative parameters were assessed.

**Results:**

Thirty-one breast lesions were verified by pathologic results, 14 of which were benign and 17 were malignant. Benign lesions were associated with dot-like, line-like, or branch-like patterns (93% vs 6%), whereas malignant lesions were associated with chaotic patterns (94% vs 7%) (*p* < 0.001). The microvasculature morphology had an area under the curve (AUC) of 0.935, a sensitivity of 94.1%, and a specificity of 92.9%. The microvasculature density, mean diameter, largest diameter, and max tortuosity of malignant lesions were significantly greater than those of benign lesions (*p* < 0.05, *p* < 0.001, *p* < 0.001, *p* < 0.05). The microvasculature mean flow velocity of benign lesions was significantly greater than that of malignant lesions (*p* < 0.05). For the quantitative parameters, the AUC was highest for the microvasculature's largest diameter (0.962), with a sensitivity of 88.2% and a specificity of 92.9%. The intra- and inter-operator reliabilities of quantitative parameters were excellent (ICC greater than 0.90).

**Conclusions:**

ULM is useful for distinguishing benign from malignant breast lesions. ULM can offer a new diagnostic method for breast lesions, which deserves further research.

**Critical relevance statement:**

This study suggests that ULM is a new technology with super-resolution that is helpful for distinguishing benign from malignant breast lesions.

**Trial registration:**

ChiCTR, ChiCTR2100048361. Registered 6 July 2021, https://www.chictr.org.cn/.

**Key Points:**

- ULM is an emerging technology that can detect highly detailed networks of microvasculature.
- Microvasculature morphology based on ULM can be a good indicator for the differential diagnosis of breast lesions.
- Among quantitative parameters extracted from ULM, microvasculature largest diameter was the best for the classification of breast lesions.

**Graphical Abstract:**

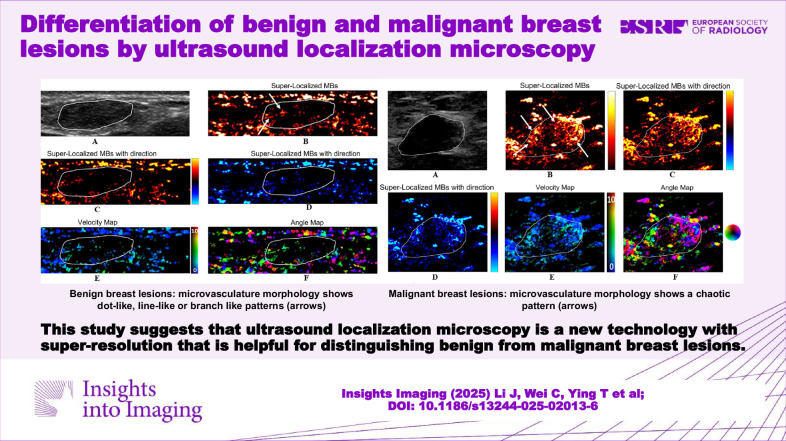

## Introduction

Breast carcinoma is the most frequent malignant tumor affecting women worldwide. Angiogenesis, which is a highly dynamic and complicated pathologic process, plays an important role in the growth and distant metastasis of tumors [1, 2]. The microvasculature contains important information on tumor angiogenesis. Compared with benign lesions, the microvasculature of malignant breast lesions exhibits a tortuous course and chaotic arrangement with irregular branches [3]. In addition, microvasculature density is a commonly used method to quantify angiogenesis in breast lesions. The microvasculature density differs between benign and malignant breast lesions. As previously reported, malignant breast lesions have higher microvasculature density than benign lesions [4]. Therefore, the analysis of microvasculature is important for the differential diagnosis of breast lesions.

To evaluate the features of vessels in breast lesions, two diagnostic imaging methods, magnetic resonance imaging (MRI) and ultrasound (US), have been widely utilized in clinical settings. Breast MRI is highly sensitive and excels in providing detailed visualization of both superficial and deeper tissues [5, 6]. Nevertheless, it offers only broad generalities regarding the microvasculature instead of depicting the detailed structure in detail because of its limited resolution. For US imaging, Doppler techniques are commonly employed to evaluate tumor vascularity. However, Doppler techniques are insensitive to low-speed blood flow [7], resulting in the loss of microvascular information. Contrast-enhanced ultrasound (CEUS) is another imaging technique that is routinely applied for the perfusion of tissue by using microbubble (MB) contrast agents, which allows the observation of the microvasculature. However, CEUS is limited in its ability to observe the microvasculature because its resolution is confined by the fundamental diffraction limit [8]. Therefore, it is also difficult to visualize the fine microvascular structure.

To overcome this limitation, ultrasound localization microscopy (ULM), also named ultrasound super-resolution microcirculation imaging (USRmi) and super-resolution ultrasound (SRUS) imaging, has been presented. ULM needs to be performed based on CEUS, which is accomplished by precisely locating individual MB contrast agents and tracking their displacements from a sequence of CEUS images [9–11]. Compared with conventional US modes, ULM can overcome the acoustic diffraction limit and show a tenfold improvement in imaging resolution [10, 11]. The resolution can reach tens of micrometers [12–14]. Therefore, ULM yields highly detailed networks of microvasculature. Moreover, hemodynamic parameters can be extracted from ULM, such as microvasculature density, direction, and velocity, which can provide more information about the microvasculature to help distinguish benign from malignant breast lesions.

Recently, ULM has gained increasing popularity. This technology has been extensively applied for imaging microvascular circulation in various animals [10, 11, 15–23]. ULM has been less studied in humans than in animals. For human breast lesions, the majority of studies have focused on visualizing the microvasculature in malignant lesions [6, 24, 25]. To date, only one study has been conducted on the classification of benign and malignant breast lesions, demonstrating that quantitative parameters, i.e., microvasculature density and microvasculature flow velocity, are helpful for differentiating breast lesions [26]. However, the role of qualitative and other quantitative parameters based on ULM remains unclear.

Therefore, we investigated the role of ULM qualitative and quantitative parameters in distinguishing benign from malignant breast lesions.

## Materials and methods

### Patients

**Fig. 1:**
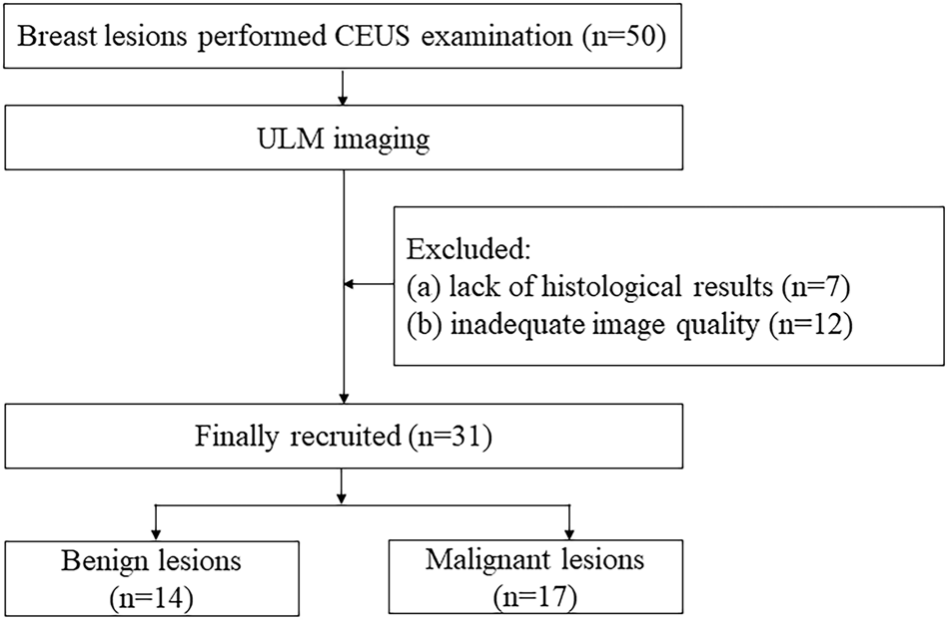
A flow diagram for the patients

We conducted a prospective and single-center study. Consecutive patients with breast lesions detected by grayscale US were recruited for CEUS examination from August 2021 to January 2023. CEUS was performed on the patients. ULM imaging was performed on CEUS video clips of breast lesions. The inclusion criteria were (a) age ≥ 18-years-old, and (b) CEUS examination prior to biopsy or surgery. The exclusion criteria included (a) a lack of histological results, and (b) inadequate image quality. A flow diagram for the patients is presented in Fig. 1.

### Conventional US and CEUS examinations

Conventional US and CEUS examinations were all performed with Philips EPIQ Elite (Philips Healthcare, Bothell, WA, USA) using an eL18-4 (4–18 MHz) linear transducer or Resona 9 (Mindray, Shenzhen, China) using a L11-3U (11 MHz) or L9-3U (9 MHz) linear transducer. Conventional US and CEUS examinations were all performed by one sonographer (sonographer 1) with 8 years of experience in breast US. The patient was in the supine position. Grayscale US was initially performed to scan the breast tumor. Color Doppler flow imaging (CDFI) was subsequently performed in different planes to evaluate intra- and extratumoral vascularity. Static images of the lesions were stored. Finally, the section with the highest number of vessels was selected for CEUS according to CDFI. We placed the transducer on the skin without pressure to avoid tissue compression during conventional US and CEUS examinations and to avoid collapsing vessel lumens. Additionally, the patient was asked to remain motionless and breathe calmly. During CEUS, the machine parameters were adjusted to the optimal parameters for each tumor; the mechanical index was less than 0.1, and the frame rate was 23–32 frames/s, affected by the depth of the examination window. Once set, no parameters were changed during the examination for each patient. CEUS was performed using the second-generation contrast agent SonoVue (Bracco, Milan, Italy). Each patient received a bolus injection of 0.5 mL of contrast agent into the forearm vein within 2 s, followed by a 5 mL saline flush. The timer on the machine started simultaneously, and the video clips were recorded for approximately 3 min from the start of the injection.

### ULM imaging and evaluation

**Fig. 2:**
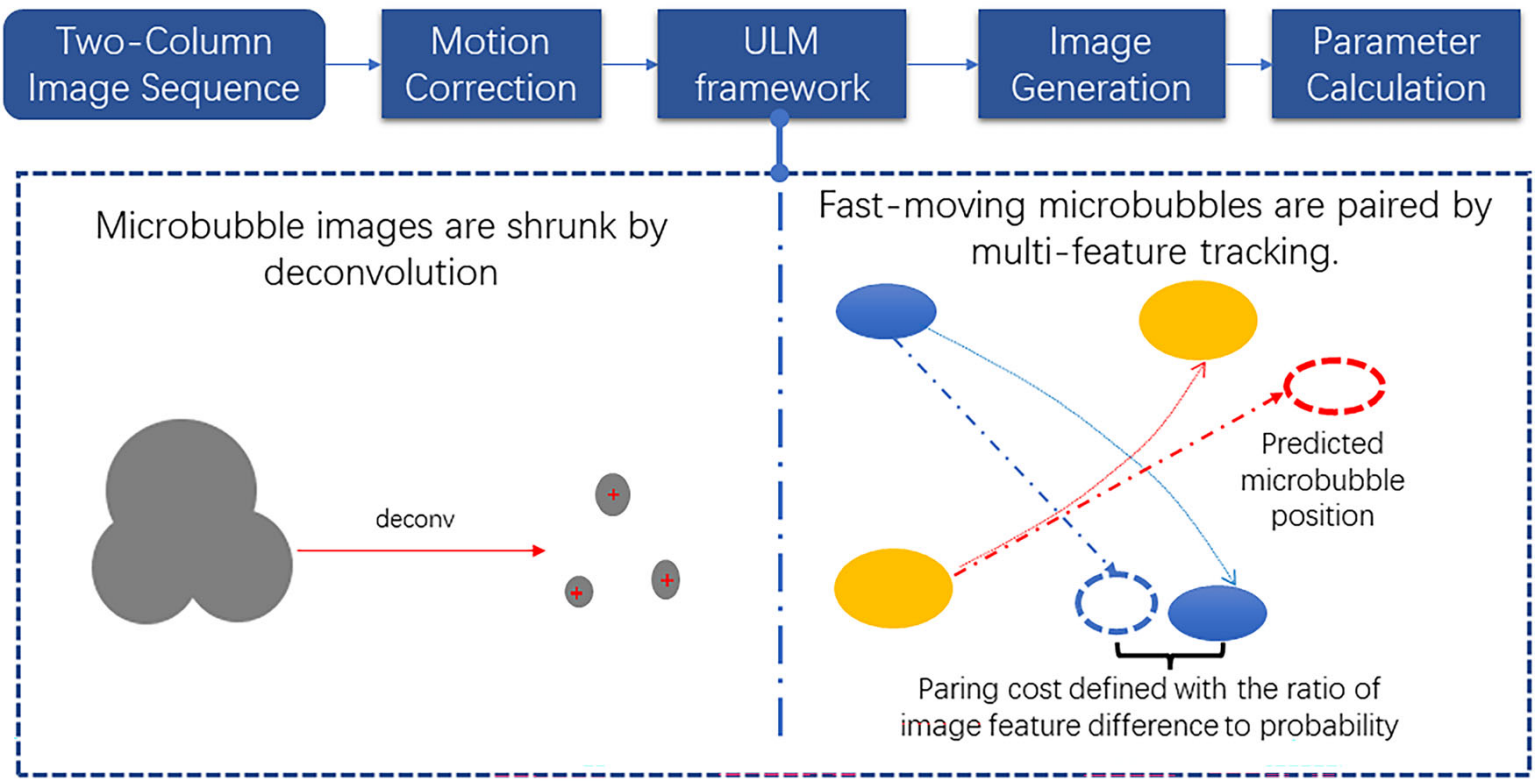
A schematic diagram of ULM. Pipeline of ULM processing. Tissue motions in the image sequence are corrected by image registration. MBs are localized with sparsity-based deconvolution. MBs in two frames are paired by finding the total minimum of the cost via graph-based assignment. A linear Kalman motion model is used to predict the MB positions and calculate the probability of two candidate-pair MBs. Then, ULM images are generated with the trajectories of MBs, and finally, metrics are calculated from the trajectories and images

The B-mode and corresponding CEUS video clips of breast lesions were postprocessed by software developed in MATLAB (R2021a, MathWorks, Natick, MA, UAS). Tissue motions, including breathing, were detected by image registration between B-mode images and corrected for the corresponding CEUS images. MBs in the CEUS sequence were localized and tracked by a framework described in a previous work that can deal with the low frame rate available in the commercial machine [27]. In the framework, sparsity-based deconvolution was able to isolate MBs from their overlapped images, and multi-feature, including MB image features and Kalman motion model, was used to pair MBs that move with large distances between consecutive frames. Super-resolution MB density map and density map with blood flow directions colored in red (toward the US transducer) and blue (going away from the US transducer) were generated by accumulating the MB trajectories. The flow velocity magnitude map and flow angle map were generated by averaging the velocity vector of trajectories on each pixel. The density maps and flow maps were blurred by two-dimensional Gaussian filtering or disk smoothing to take into account the localization uncertainty in the image reconstruction. A schematic diagram of ULM is presented in Fig. 2.

The qualitative and quantitative parameters of breast lesions extracted from the ULM images were recorded. The qualitative parameter was microvasculature morphology. Microvasculature morphology was divided into two types: (1) dot-like, line-like, or branch-like patterns and (2) chaotic patterns. Dot-like, line-like, or branch-like patterns were defined as dots, straight or curved vessels, with or without branching vessels, and with or without peripheral annular vessels. A chaotic pattern was defined as a disordered arrangement of vessels that formed an abnormal vascular network. The quantitative parameters included microvasculature density, diameter (mean diameter and largest diameter), tortuosity (mean tortuosity and max tortuosity), and flow velocity (mean flow velocity and max flow velocity). The microvascular diameter was measured with a micrometer.

The two trained sonographers (sonographer 2 and sonographer 3) independently performed ULM imaging and reviewed ULM qualitative parameters of each lesion. If the final results were not uniform, the ULM qualitative parameters were assessed by Sonographer 4. All sonographers were blinded to the results of pathology, clinical histology, and other examinations. In addition, ULM quantitative parameters were recorded by Sonographer 2 after performing ULM imaging. To test intra-operator reliabilities for quantitative parameters, Sonographer 2 repeated the operation after 1 month. To test inter-operator reliabilities for quantitative parameters, Sonographer 3 performed a second operation. Sonographers 2 and 3 were blinded to each other’s results.

### Statistical analysis

The Shapiro–Wilk test was used to check whether the data were normally distributed. Continuous variables were presented as the mean ± standard deviation (SD) or median (P25, P75). Categorical variables were presented as frequencies and percentages. To compare ULM qualitative parameters, Fisher’s exact test was employed. To compare ULM quantitative parameters, an independent sample *t*-test or a Mann–Whitney *U*-test was used. A receiver operating characteristic (ROC) curve was applied to assess the diagnostic performance of the ULM qualitative and quantitative parameters. The area under the curve (AUC), sensitivity, and specificity were computed. The optimal cut-off value for quantitative parameters was obtained by ROC curve analysis, which maximized the Youden index. AUC, sensitivity, and specificity were calculated using a 95% confidence interval (CI). The intra- and inter-operator reliabilities of the quantitative parameters were assessed by the interclass correlation coefficient (ICC). Significance was indicated by a *p* value < 0.05. Statistical analysis was performed using SPSS and MedCalc.

## Results

### Clinical characteristics

All the patients were females (mean age: 51.54 ± 17.28 years, range: 23–81 years). Forty-one patients with 50 breast lesions underwent CEUS examination in this study. Ultimately, 24 patients with 31 breast lesions were included in this study. Thirty-one breast lesions were verified by pathologic results of surgical resection or needle biopsy, 14 of which were benign and 17 were malignant. Table 1 summarizes the pathologic results of breast lesions.

**Table 1:** Pathologic results of breast lesions (*n* = 31)

Breast tumors	Number/percentage
Benign	14 (45%)
Fibroadenoma	8
Intraductal papilloma	2
Adenosis	3
Benign phyllodes tumor	1
Malignant	17 (55%)
Invasive breast carcinoma	14
Ductal carcinoma in situ	2
Mucinous carcinoma	1

### Comparison of benign and malignant breast lesions based on ULM qualitative parameters

**Fig. 3:**
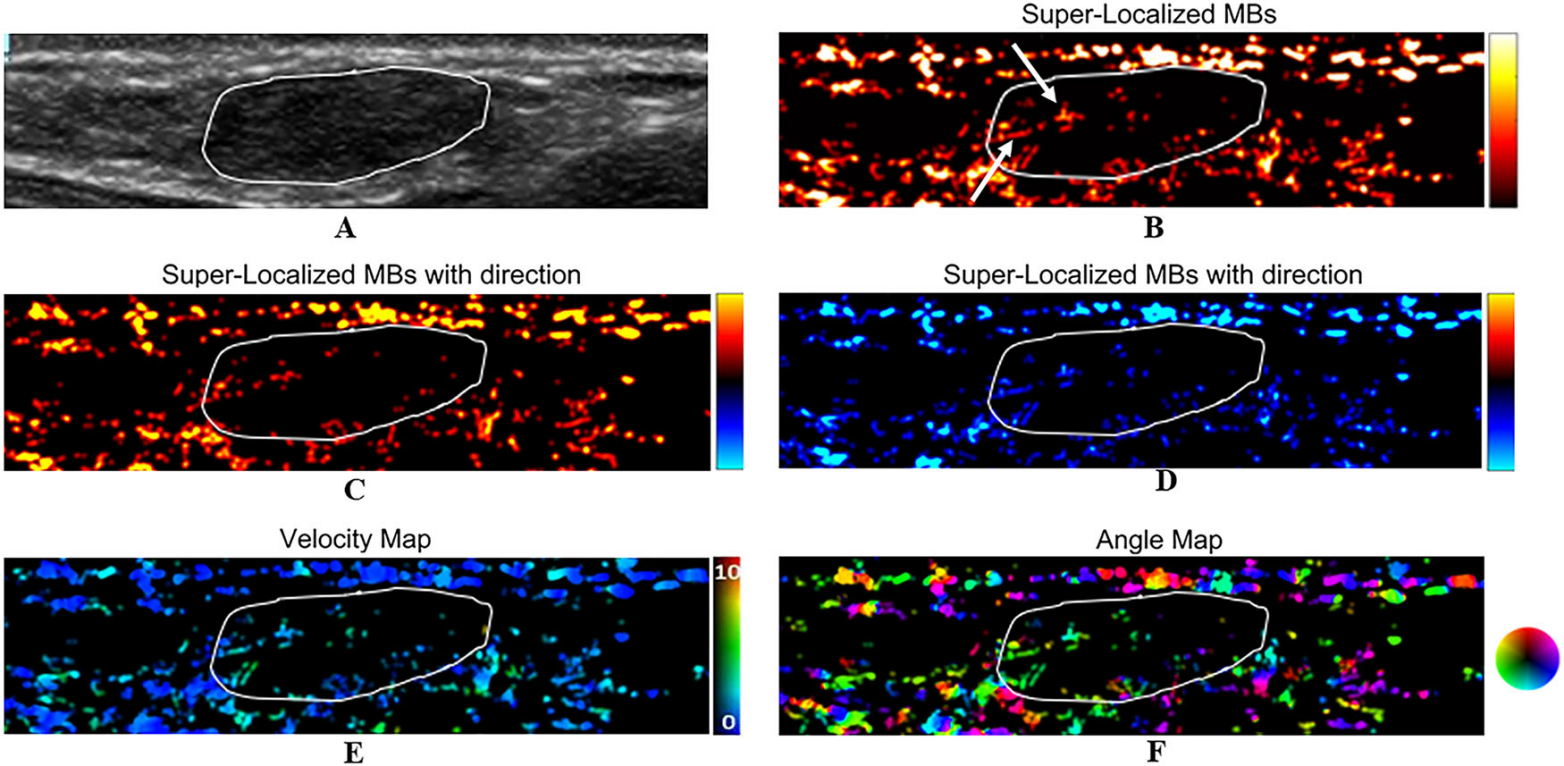
A 30-year-old woman with a breast fibroadenoma. The tumor region is marked in white. **A** Grayscale US image shows a breast lesion. **B** ULM microvasculature localized density map. The microvasculature morphology shows dot-like, line-like, or branch-like patterns (arrows). **C** ULM microvasculature direction map. The red color shows blood flow toward the US transducer. **D** ULM microvasculature direction map. The blue color shows blood flow going away from the US transducer. **E** ULM microvasculature velocity magnitude map, with the color map showing the magnitude of the velocity. **F** ULM microvasculature angle map

**Fig. 4:**
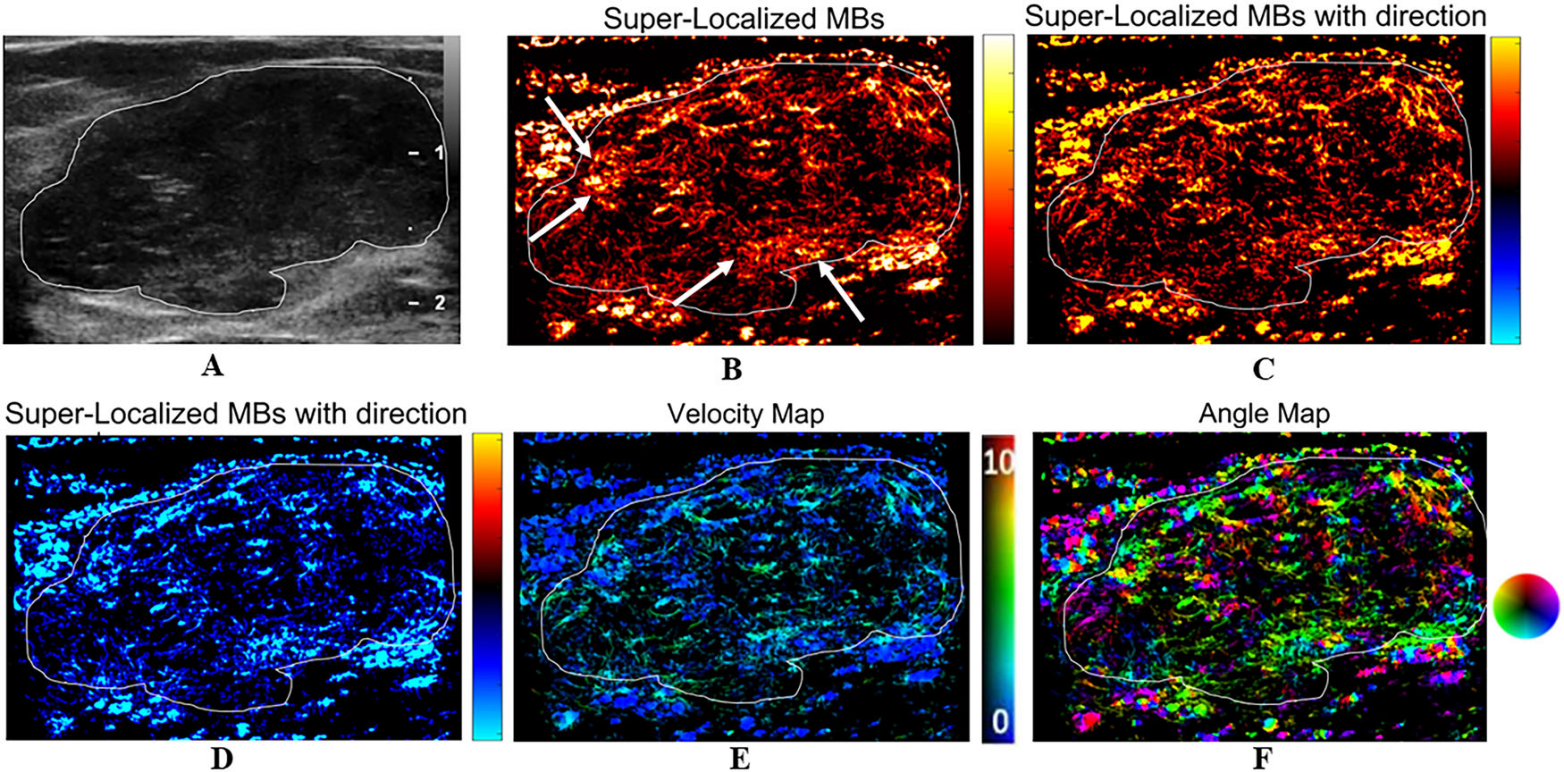
A 52-year-old woman with a benign PT of the breast. The tumor region is marked in white. **A** Grayscale US image shows a breast lesion. **B** ULM microvasculature localized density map. The microvasculature morphology shows a chaotic pattern (arrows). **C** ULM microvasculature direction map. The red color shows blood flow toward the US transducer. **D** ULM microvasculature direction map. The blue color shows blood flow going away from the US transducer. **E** ULM microvasculature velocity magnitude map, with the color map showing the magnitude of the velocity. **F** ULM microvasculature angle map

**Fig. 5:**
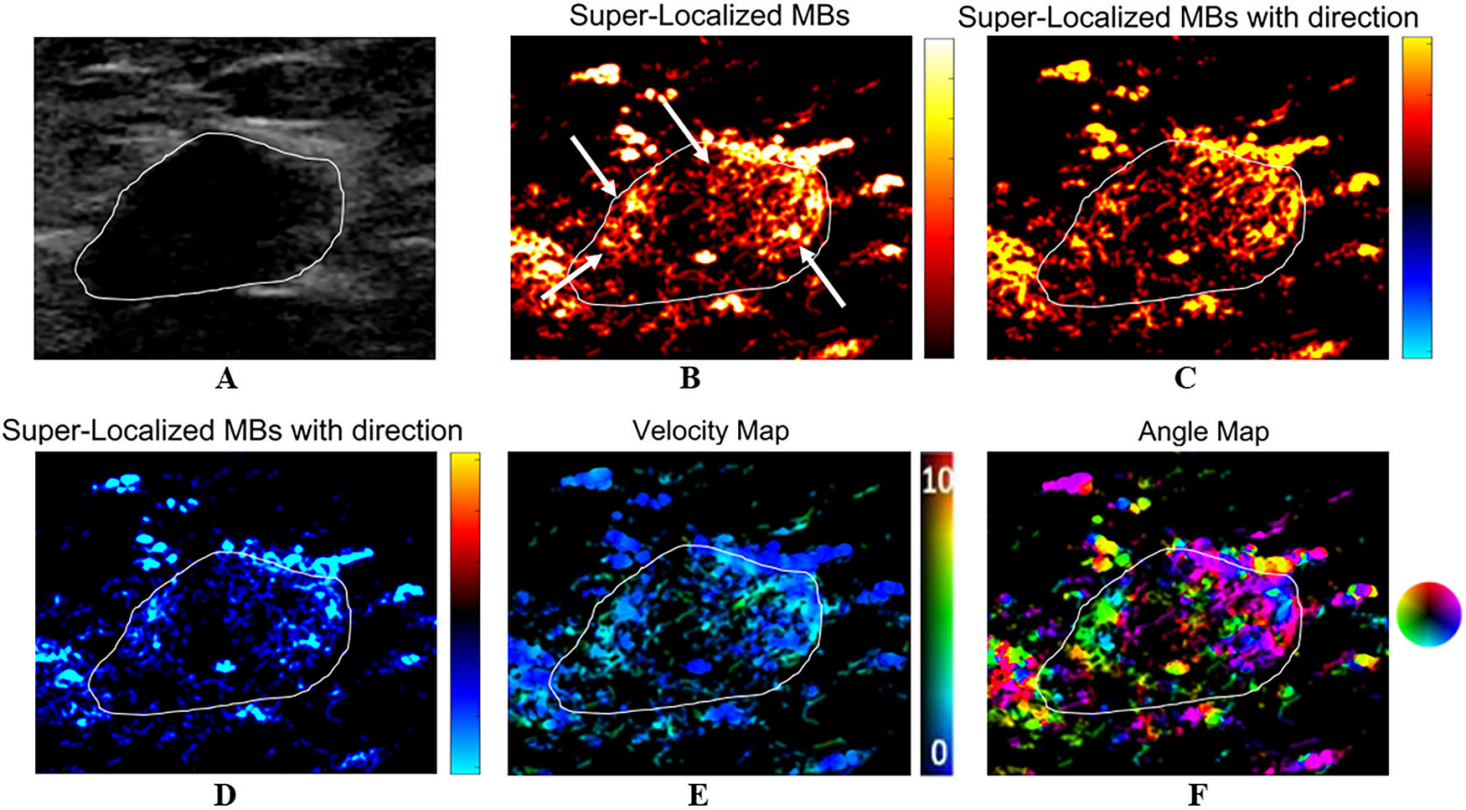
A 73-year-old woman with invasive breast carcinoma. The tumor region is marked in white. **A** Grayscale US image shows a breast lesion. **B** ULM microvasculature localized density map. The microvasculature morphology shows a chaotic pattern (arrows). **C** ULM microvasculature direction map. The red color shows blood flow toward the US transducer. **D** ULM microvasculature direction map. The blue color shows blood flow going away from the US transducer. **E** ULM microvasculature velocity magnitude map, with the color map showing the magnitude of the velocity. **F** ULM microvasculature angle map

With respect to microvasculature morphology, benign lesions were associated with dot-like, line-like, or branch-like patterns (93% vs 6%), whereas malignant lesions were associated with chaotic patterns (94% vs 7%) (*p* < 0.001) (Table 2). Among the benign lesions, a chaotic pattern was present in 1 case of a benign phyllodes tumor (PT). In addition, there were 6 cases of benign lesions that were characterized as malignant lesions on CEUS, whereas ULM revealed a benign appearance, including 2 cases of fibroadenoma, 1 case of intraductal papilloma, and 3 cases of adenosis. Figures 3–5 show the ULM features of benign and malignant lesions.

**Table 2:** Comparison of benign and malignant breast lesions based on qualitative parameters

Pathology	Dot-like, line-like, or branch-like patterns [*n* (%)]	Chaotic patterns [*n* (%)]
Benign (*n* = 14)	13 (93%)	1 (7%)
Malignant (*n* = 17)	1 (6%)	16 (94%)
*p* value	< 0.001^a,*^	

^a^ Fisher’s exact test

^*^ Statistically significant

### Comparison of benign and malignant breast lesions based on ULM quantitative parameters

The microvasculature density [46.00 (23.50, 70.00) % vs 12.00 (8.75, 26.00) %, *p* < 0.05], mean diameter [254.89 (231.32, 314.76) μm vs 127.49 (112.79, 148.45) μm, *p* < 0.001], largest diameter [1341.86 (965.38, 1591.64) μm vs 348.94 (287.54, 460.63) μm, *p* < 0.001] and max tortuosity [46.48 (34.21, 68.45) vs 24.36 (22.43, 53.17), *p* < 0.05] of malignant lesions were significantly greater than those of benign lesions. The microvasculature mean flow velocity [(2.02 ± 0.30) mm/s vs (1.62 ± 0.47) mm/s, *p* < 0.05] of benign lesions was significantly greater than that of malignant lesions. However, there were no significant differences in the microvasculature mean tortuosity [2.54 (2.45, 3.20) vs 2.54 (2.28, 2.71), *p* > 0.05] or max flow velocity [9.00 (8.07, 9.26) mm/s vs 9.41 (7.73, 9.71) mm/s, *p* > 0.05] between benign and malignant lesions (Table 3).

**Table 3:** Comparison of benign and malignant breast lesions based on ULM quantitative parameters

Parameters	Benign (*n* = 14)	Malignant (*n* = 17)	*p* value
Microvasculature density (%), median (P25, P75)	12.00 (8.75, 26.00)	46.00 (23.50, 70.00)	0.001^a,*^
Microvasculature diameter (μm)			
Mean diameter, median (P25, P75)	127.49 (112.79, 148.45)	254.89 (231.32, 314.76)	< 0.001^a,*^
Largest diameter, median (P25, P75)	348.94 (287.54, 460.63)	1341.86 (965.38, 1591.64)	< 0.001^a,*^
Microvasculature tortuosity			
Mean tortuosity, median (P25, P75)	2.54 (2.45, 3.20)	2.54 (2.28, 2.71)	0.159^a^
Max tortuosity, median (P25, P75)	24.36 (22.43, 53.17)	46.48 (34.21, 68.45)	0.032^a,*^
Microvasculature flow velocity (mm/s)			
Mean flow velocity, mean ± SD	2.02 ± 0.30	1.62 ± 0.47	0.009^b,*^
Max flow velocity, median (P25, P75)	9.00 (8.07, 9.26)	9.41 (7.73, 9.71)	0.153^a^

*SD* standard deviation

^a^ Mann–Whitney *U*-test

^b^ Independent sample *t*-test

^*^ Statistically significant

### Diagnostic performance of ULM qualitative and quantitative parameters between benign and malignant breast lesions

**Fig. 6:**
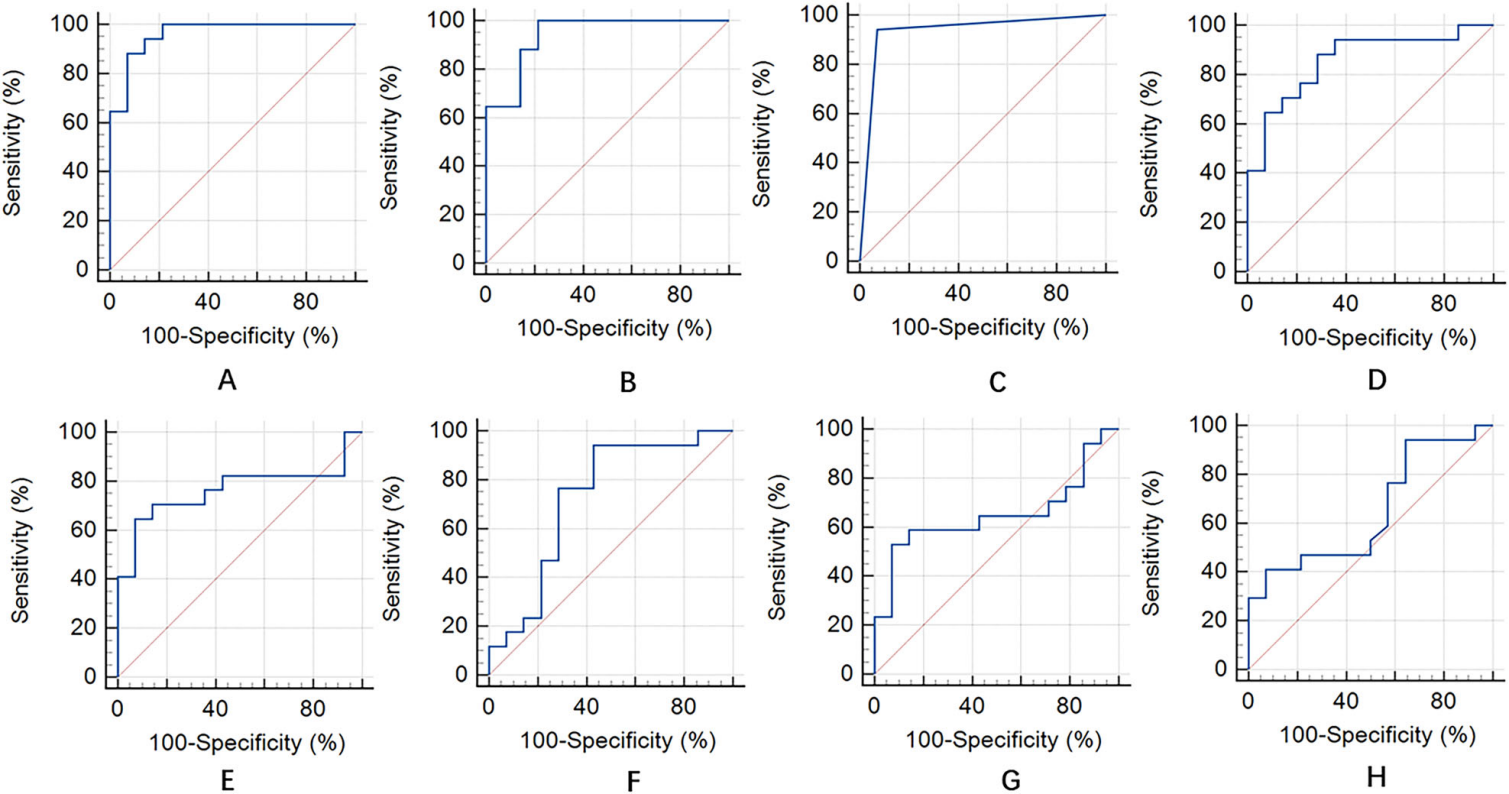
ROC curves of the ULM qualitative and quantitative parameters for the differentiation of benign and malignant breast lesions. The AUCs of the qualitative and quantitative parameters were as follows: **A** microvasculature largest diameter: 0.962 (95% CI: 0.824–0.998); **B** microvasculature mean diameter: 0.941 (95% CI: 0.794–0.994); **C** microvasculature morphology: 0.935 (95% CI: 0.785–0.992); **D** microvasculature density: 0.857 (95% CI: 0.685–0.956); **E** microvasculature mean flow velocity: 0.765 (95% CI: 0.579–0.898); **F** microvasculature max tortuosity: 0.727 (95% CI: 0.538–0.871); **G** microvasculature max flow velocity: 0.651 (95% CI: 0.460–0.813); and **H** microvasculature mean tortuosity: 0.649 (95% CI: 0.458–0.811)

The microvasculature morphology had an AUC of 0.935 (95% CI: 0.785–0.992), a sensitivity of 94.1%, and a specificity of 92.9% for the differentiation of benign and malignant lesions. With respect to the quantitative parameters, the AUC was highest for the microvasculature largest diameter (0.962, 95% CI: 0.824–0.998), followed by the microvasculature mean diameter (0.941, 95% CI: 0.794–0.994), density (0.857, 95% CI: 0.685–0.956), mean flow velocity (0.765, 95% CI: 0.579–0.898), max tortuosity (0.727, 95% CI: 0.538–0.871), max flow velocity (0.651, 95% CI: 0.460–0.813) and mean tortuosity (0.649, 95% CI: 0.458–0.811) for the differentiation of benign and malignant lesions. At a cut-off of 763.88 μm, microvasculature largest diameter had a sensitivity of 88.2% and a specificity of 92.9% for the differentiation of benign and malignant lesions (Table 4). Figure 6 shows the ROC curves of the ULM qualitative and quantitative parameters for the differentiation of benign and malignant breast lesions.

**Table 4:** Diagnostic performance of ULM qualitative and quantitative parameters for benign and malignant breast lesions

Parameters	Sensitivity % (95% CI)	Specificity % (95% CI)	Cut-off
Qualitative			
Microvasculature morphology	94.1 (71.3–99.9)	92.9 (66.1–99.8)	–
Quantitative			
Microvasculature density (%)	88.2 (63.6–98.5)	71.4 (41.9–91.6)	18
Microvasculature diameter (μm)			
Mean diameter	100 (80.5–100.0)	78.6 (49.2–95.3)	137.98
Largest diameter	88.2 (63.6–98.5)	92.9 (66.1–99.8)	763.88
Microvasculature tortuosity			
Mean tortuosity	41.2 (18.4–67.1)	92.9 (66.1–99.8)	2.33
Max tortuosity	94.1 (71.3–99.9)	57.1 (28.9–82.3)	24.78
Microvasculature flow velocity (mm/s)			
Mean flow velocity	64.7 (38.3–85.8)	92.9 (66.1–99.8)	1.65
Max flow velocity	52.9 (27.8–77.0)	92.9 (66.1–99.8)	9.33

*CI* confidence interval

### Intra- and inter-operator reliabilities of the quantitative parameters

The intra- and inter-operator reliabilities of the quantitative parameters were excellent, with ICCs greater than 0.900. For intra-operator reliability, the ICCs of the microvasculature density, mean diameter, largest diameter, mean tortuosity, max tortuosity, mean flow velocity, and max flow velocity were 1.000, 0.999, 0.999, 0.994, 0.964, 0.999, and 0.998, respectively. For inter-operator reliability, the ICCs of the microvasculature density, mean diameter, largest diameter, mean tortuosity, max tortuosity, mean flow velocity, and max flow velocity were 0.902, 0.968, 0.998, 0.995, 0.947, 0.999, and 0.984, respectively (Table 5).

**Table 5:** The intra- and inter-operator reliabilities of the quantitative parameters

Parameters	intra-operator ICC (95% CI)	inter-operator ICC (95% CI)
Microvasculature density	1.000 (0.999–1.000)	0.902 (0.808–0.951)
Mean diameter	0.999 (0.998–1.000)	0.968 (0.935–0.984)
Largest diameter	0.999 (0.998–0.999)	0.998 (0.997–0.999)
Mean tortuosity	0.994 (0.987–0.997)	0.995 (0.989–0.997)
Max tortuosity	0.964 (0.927–0.983)	0.947 (0.893–0.974)
Mean flow velocity	0.999 (0.998–1.000)	0.999 (0.998–1.000)
Max flow velocity	0.998 (0.995–0.999)	0.984 (0.967–0.992)

*CI* confidence interval

## Discussion

In the present study, the role of ULM qualitative and quantitative parameters in distinguishing benign from malignant breast lesions was evaluated. Our results revealed that dot-like, line-like, or branch-like patterns were significantly correlated with benign breast lesions and that chaotic patterns were significantly correlated with malignant breast lesions regarding qualitative parameters. With respect to quantitative parameters, the microvasculature density, mean diameter, largest diameter, and max tortuosity of malignant lesions were significantly greater than those of benign lesions; the microvasculature mean flow velocity of benign lesions was significantly greater than that of malignant lesions. Among quantitative parameters, the AUC was highest for the microvasculature’s largest diameter for the differentiation of breast lesions.

To date, the microvasculature morphology of ULM has not been studied in human breast lesions. Other US imaging techniques, such as CDFI, power Doppler imaging (PDI), superb microvascular imaging (SMI), and CEUS, have reported the value of vessel morphology in the differential diagnosis of breast lesions. However, the findings varied between studies. Gokalp et al [28] demonstrated that a line-like pattern was associated with benign breast lesions and that a branch-like or chaotic pattern was associated with malignant breast lesions using PDI. Diao et al [29] reported that a line-like pattern was present mainly in benign breast lesions, and a branch-like pattern was also present mainly in benign breast lesions with SMI and CEUS. They also revealed that vessel morphology was not significantly different between benign and malignant breast lesions according to the CDFI and PDI. Our study revealed that dot-like, line-like, or branch-like patterns were significantly correlated with benign breast lesions and that chaotic patterns were significantly correlated with malignant breast lesions using ULM. One possible explanation for the discrepancy in vessel morphology in different studies is the difference in US imaging techniques, resulting in different sensitivities to vessels. We further assessed the diagnostic performance of microvasculature morphology and found that it achieved a relatively high AUC (0.935), sensitivity (94.1%), and specificity (92.9%). These findings suggest that microvasculature morphology based on ULM can be a good indicator for the differential diagnosis of breast lesions.

Compared with conventional US imaging techniques, quantitative parameters based on ULM can not only obtain information at the microvasculature level but also directly compute these parameters. We evaluated seven quantitative parameters, including microvasculature density, mean diameter, largest diameter, mean tortuosity, max tortuosity, mean flow velocity, and max flow velocity. Among them, microvasculature density has become the gold standard for evaluating tumor angiogenesis [30]. In the present study, the microvasculature density of malignant breast lesions was significantly greater than that of benign lesions (*p* < 0.05). The result was in agreement with that of a previous study [26]. In fact, malignant breast lesions often exhibit hypervascularity [31, 32]. Thus, it is not surprising that malignant lesions have higher microvasculature density than benign lesions.

In this study, we found that the microvasculature’s largest diameter and mean diameter in malignant breast lesions were significantly greater than those of benign lesions (all *p* < 0.001). This can be attributed to the fact that dilated large vessels are commonly observed in malignant breast lesions [33]. These results were in line with those of a previous study by Gu et al [34] in which US high-definition microvasculature imaging was used. However, compared with that study, our study was superior in terms of the precision of microvascular measurement, as the microvascular diameter was measured on a micrometer scale. In that study, the microvascular diameter was measured in millimeters. In other words, ULM can offer more information on the microvasculature. Additionally, the diagnostic performance of the microvasculature's largest diameter and mean diameter was first evaluated in this study. We found that the AUC of microvasculature largest diameter was greater than that of microvasculature mean diameter (0.962 vs 0.941), suggesting that the diagnostic value of microvasculature largest diameter was better than that of microvasculature mean diameter.

Vessel tortuosity is used to assess the degree of vessel bending, which is likely correlated with growth factor-related alterations in the vessel wall, such as epithelial cell proliferation, changes in the basement membrane, and loss of pericytes and smooth muscle [35]. Other US imaging techniques have revealed that an increase in vessel tortuosity is significantly associated with malignant breast lesions [36, 37]. Ternifi et al [37] investigated microvasculature tortuosity, including microvasculature mean tortuosity and max tortuosity, using US high-definition microvasculature imaging and found that the microvasculature max tortuosity of malignant breast lesions was greater than that of benign lesions, but the microvasculature mean tortuosity was not significantly different. These findings are consistent with those in our study. However, our study was the first to evaluate the diagnostic performance of microvasculature max tortuosity and mean tortuosity. Similar to microvasculature diameter, the AUC of microvasculature max tortuosity was also greater than that of microvasculature mean tortuosity (0.727 vs 0.649), suggesting that the diagnostic value of microvasculature max tortuosity was better than that of microvasculature mean tortuosity. Based on the above results, more consideration needs to be given to the max values of microvasculature diameter and tortuosity.

Microvasculature flow velocity is another quantitative parameter obtained by ULM. In clinical practice, vessel flow velocity is often detected by Doppler US. However, Doppler US only detects fast flow (greater than 1 cm/s) [6]. In contrast, ULM is a new imaging method that has high sensitivity for detecting low flow. Furthermore, ULM can clearly visualize the magnitude of the microvasculature flow velocity of breast lesions by a velocity map. Evaluating the blood flow status of tumors accurately can help identify malignant tumors [38, 39]. Max flow velocity has been reported to be associated with the protein expression of genes related to angiogenesis in breast cancer. Niu et al [40] reported that the protein expression of VEDGF165, NRP-1, SphK1, CD31, YAP, CTGF, and Gli2 was positively correlated with the max flow velocity; however, the protein expression of PTEN and MFN2 was negatively correlated with the max flow velocity. Our study showed that the microvasculature max flow velocity was not significantly different for the classification of breast lesions (*p* > 0.05), which was inconsistent with the findings of a previous study [26]. Unlike the max flow velocity, the microvasculature mean flow velocity of benign breast lesions was significantly greater than that of malignant lesions (*p* < 0.05). To date, no studies have explored the diagnostic value of the microvasculature mean flow velocity based on US imaging between benign and malignant breast lesions in humans.

PTs and fibroadenomas of the breast need to be distinguished because they have similar features in terms of histopathology, clinical findings, and radiological findings [41]. Clinically, the approach to PTs and fibroadenomas includes different treatment modalities. In the US, classification of PTs and fibroadenomas is challenging because substantial overlap exists between the two diseases [42, 43]. In the present study, there was 1 case of benign PT and 8 cases of fibroadenoma. These lesions had different microvasculature morphologies on ULM. Benign PTs exhibited a chaotic pattern, whereas fibroadenomas exhibited dot-like, line-like, or branch-like patterns. These findings indicate that ULM could provide a new method for differentiating between benign PTs and fibroadenomas.

ULM, with the ability to provide high spatial resolution for visualizing microvasculature, has widespread potential clinical applications for managing breast diseases. For example, ULM can identify the richest area of vessels, which could be used to increase the accuracy of biopsy and decrease biopsy rates. Furthermore, ULM has the greatest value in appropriately identifying low-suspicion benign lesions, which have a high rate of misdiagnosis as malignant lesions on CEUS. In our study, six lesions were characterized as malignant lesions via CEUS, whereas ULM revealed a benign appearance. Therefore, ULM has the potential to reduce unnecessary biopsies of benign lesions. In addition, the intra- and inter-operator reliabilities of ULM quantitative parameters were excellent (ICC greater than 0.90) in our study. It is indicated that ULM is reproducible. However, ULM has several limitations. It is affected by the MB concentration and tissue motion, which play significant roles in localization accuracy. A high concentration of MB makes it difficult to identify each MB in successive frames [44]. For tissue motion, mild in-plane motion can be corrected, but relatively large motion is difficult to correct [8]. In this study, the reason for the suboptimal imaging was the high concentration of MB and the large motion due to patient cooperation. In this case, alternative techniques, such as CEUS and contrast-free super-resolution power Doppler (CS-PD) based on deep neural networks, can be considered for evaluating the microvasculature [45].

Our study had several limitations. First, a larger sample size is necessary, especially for analyzing diagnostic performance. Although 31 lesions were ultimately included in this study, the number of samples remained small, and a larger sample size will be necessary. Second, we performed the study with 2-dimensional US, which may not fully reflect the vessels of the whole lesion in comparison with 3-dimensional US. To obtain more vessels, we selected the plane with the richest density of vessels to perform CEUS examination and consequently to perform ULM.

In conclusion, ULM is useful for distinguishing benign from malignant breast lesions. ULM can offer a new diagnostic method for breast lesions, which deserves further research.

## Data Availability

All data used or analyzed in this study are available from the corresponding author upon reasonable request.

## Abbreviations

AUC: Area under the curve
CDFI: Color Doppler flow imaging
CEUS: Contrast-enhanced ultrasound
CI: Confidence interval
MB: Microbubble
MRI: Magnetic resonance imaging
PDI: Power Doppler imaging
PT: Phyllodes tumor
ROC: Receiver operating characteristic
SD: Standard deviation
SMI: Superb microvascular imaging
ULM: Ultrasound localization microscopy
US: Ultrasound
